# Polymeric additives to enhance the functional properties of calcium phosphate cements

**DOI:** 10.1177/2041731412439555

**Published:** 2012-03-20

**Authors:** Roman A Perez, Hae-Won Kim, Maria-Pau Ginebra

**Affiliations:** 1 Biomaterials, Biomechanics, and Tissue Engineering Group, Department of Materials Science and Metallurgy, Technical University of Catalonia (UPC), Barcelona, Spain; 2 Biomedical Research Networking Center in Bioengineering, Biomaterials and Nanomedicine (CIBER-BBN), Zaragoza, Spain; 3 Institute of Tissue Regeneration Engineering (ITREN), Dankook University, Cheonan, South Korea; 4 Department of Biomaterials Science, College of Dentistry, Dankook University, Cheonan, South Korea; 5 Department of Nanobiomedical Science and WCU Research Center, Dankook University, Cheonan, South Korea

**Keywords:** calcium phosphate cement, polymer, hydroxyapatite

## Abstract

The vast majority of materials used in bone tissue engineering and regenerative medicine are based on calcium phosphates due to their similarity with the mineral phase of natural bone. Among them, calcium phosphate cements, which are composed of a powder and a liquid that are mixed to obtain a moldable paste, are widely used. These calcium phosphate cement pastes can be injected using minimally invasive surgery and adapt to the shape of the defect, resulting in an entangled network of calcium phosphate crystals. Adding an organic phase to the calcium phosphate cement formulation is a very powerful strategy to enhance some of the properties of these materials. Adding some water-soluble biocompatible polymers in the calcium phosphate cement liquid or powder phase improves physicochemical and mechanical properties, such as injectability, cohesion, and toughness. Moreover, adding specific polymers can enhance the biological response and the resorption rate of the material. The goal of this study is to overview the most relevant advances in this field, focusing on the different types of polymers that have been used to enhance specific calcium phosphate cement properties.

## Introduction

The search for new synthetic bone grafts is a topic of extensive research. Although autografts are the gold standard for targeted bone regeneration, they present some disadvantages such as pain morbidity, disease transmission, and limited availability.^1–7^ The development of synthetic materials is an alternative strategy to overcome the limitations associated with these problems. The challenge to material scientists is to produce biomaterials with properties that mimic the natural extracellular matrix of bone tissue, which is mainly composed of hydroxyapatite (HA) and collagen.^8^ Hence comes the potential for calcium phosphate-based materials, which resemble the bone mineral phase, and more specifically calcium phosphate cements (CPCs). This family of materials allows self-setting HA or brushite (dicalcium phosphate dihydrate (DCPD)) to be obtained by soft chemistry routes. The properties of these two families of cements are quite different. HA CPCs tend to be stronger. Moreover, since DCPD is metastable in physiological conditions, brushite CPCs are much faster resorbable than apatite CPCs, although it has been shown that in vivo DCPD tends to convert into precipitated hydroxylapatite (PHA). CPCs are composed of a powder phase and a liquid phase, which are mixed to form a moldable and injectable paste at a determined liquid to powder (L/P) ratio to obtain a final product different from the initial reagents. The final properties of this end product can be tailored by changing different processing parameters, such as the composition and granulometry of the powder phase, the composition of the liquid phase, or L/P used. The final CPC product arises from a dissolution–precipitation reaction, which produces hydrated compounds with a composition and morphology close to the calcium phosphates found in mineralized tissues.^9^

An approach that is attracting much attention in the CPC field is to incorporate polymers into the formulation, either as a second solid phase or dissolved in the liquid phase. This appears to be an excellent option to enhance CPC performance and improve not only some properties relevant for the clinical use of these materials, such as injectability, cohesion, or setting time, but also their final performance in terms of resorption rate and cell/tissue response.

The scope of this study is to overview the role of polymers in the design of more efficient CPC formulations. The use of different natural and synthetic polymers is reviewed, and their effects on different CPC properties are analyzed.

## Why add a polymer to a CPC?

Incorporating polymers has been a strategy to overcome the intrinsic limitations of an inorganic CPC. Many properties can be improved by adding a polymer phase. Although the effect of adding a polymer depends on the composition of the organic phase, the main trends for some relevant CPC properties are summarized in the following.

### Setting time

The setting time is the time when the CPC paste loses its plasticity and starts to harden to form a solid body. Setting times are usually measured by indentation, which is a fast and easy system, although it is imprecise. The most commonly used method consists of two Gilmore needles with different loads that may penetrate into the sample depending on the hardness of the solid paste. Once the needles do not penetrate the sample, the setting time is completed. A CPC must have appropriate setting times of 5–15 min.^10^ As a general rule, the presence of polymers tends to increase setting time, which may be related to the higher viscosity of the polymer-containing paste, which hinders ion diffusion in the matrix.

### Cohesion/washout resistance

Cohesion is the ability of a paste to set in a fluid without disintegrating. Different terms have been used to describe this property, such as nondecay ability, antiwashout, compliance, swelling, or stability, and several studies have been performed on this topic.^10–16^ Nevertheless, disintegration of the cement paste, in addition to preventing the cement from setting, can provoke an inflammatory response and cell apoptosis.^17^ For this reason, the cohesion time should be lower than the initial setting time to guarantee the structural integrity of the cement paste.^10^ In general, adding soluble polymers during the liquid phase tends to enhance CPC cohesion. The mechanism underlying this phenomenon is the increased viscosity of the CPC paste, which prevents penetration of the surrounding fluid.

### Injectability

Injectability is a CPC property most appreciated by clinicians, as it allows minimizing the surgery and permits adequate filling of complex-shaped defects. Injectability is the ability of a paste to be extruded through a needle without demixing. Injectability can be increased by increasing the CPC L/P ratio, although this adversely affects mechanical properties.^18,19^ Some water-soluble polymers, such as polysaccharides, have been extensively used to enhance CPC injectability and to increase cohesion time.

### Macroporosity

CPCs are intrinsically porous materials, with pores in the micro- or nanometer range,^20^ but lack macroporosity, which is an essential feature for tissue colonization and angiogenesis. Two main routes have been explored to introduce macroporosity into CPCs by adding polymers: (a) foaming the liquid phase or the cement paste containing a polymer,^21–25^ and (b) loading the CPC with biodegradable polymers (e.g. microspheres (MSs) or fibers) that slowly degrade over time, resulting in a macroporous structure.^26,27^ Actually, even a third method has been proposed in which a collagen and CPC slurry are freeze–dried to produce a macroporous scaffold, although this is no longer injectable.^28^

### Mechanical properties

Poor mechanical performance of CPCs has limited their applicability to nonload-bearing applications.^29^ Due to the intrinsic porosity of CPCs, their strength is lower than that of calcium phosphate ceramics. Moreover, their toughness, ductility, and fatigue resistance are much less than those of cortical bone. Incorporating a polymer during the CPC liquid phase increases ductility, allowing for a higher deformation before breaking. Moreover, polymer fiber reinforcement has been extensively explored as a strategy to increase toughness and strength of cements.^30^

### Long-term degradation

One of the main drawbacks when working with most CPCs, particularly those resulting in HA as the reaction product, is their slow resorption rate, which impairs healing. In this sense, the strategies mentioned previously aimed at creating macropores in the CPC, namely, incorporation of biodegradable polymers and foaming, also result in an increase in the degradation rate.

### Drug eluting properties

The intrinsic porosity of CPCs has been exploited for use in drug delivery applications. The combination of CPC with polymers has been used as a way to tune drug release kinetics.

### Biological response

CPCs generally have low cell attachment and low proliferation rates when cells are cultured in vitro, basically due to the spiky crystal morphology that arises from the precipitation of the initial powder.^31–36^ Therefore, incorporating some polymers may add specific binding domains to permit cell adhesion. The most well-known specific binding domains are those related to cell attachment, such as the RGD sequence found in gelatin.

The different properties that can be enhanced by the addition of the specific polymers in the CPC are listed in Table 1.

**Table 1.** Some properties of calcium phosphate cements that can be improved by the incorporation of a polymeric phase and the corresponding polymers

Property improved	Polymers associated in liquid phase	Polymers associated in powder phase
Setting time	Alginate	—
	Chitin	
	PEG	
Cohesion	Chitosan	—
	Alginate	
	Silk	
	PEG	
Injectability	Hyaluronate	—
	Cellulose	
Macroporosity	Soybean	Gelatin
	Albumen	Polyesters
Mechanical properties	Gelatin	Chitosan
	Chitosan	Polyesters
	Chitin	
	Polyesters	
	PAA	
	Fibrin glue	
Long-term degradation	—	Gelatin
		Chitosan
		Polyesters
Drug eluting system	Chitosan	Gelatin
	Polyesters	Polyesters
	PAA	
Biological response	Gelatin	Alginate
	Collagen	Polyesters

PEG: polyethylene glycol; PAA: polyacrylic acid.

## Ways of incorporating polymers to CPCs

**Figure 1.**
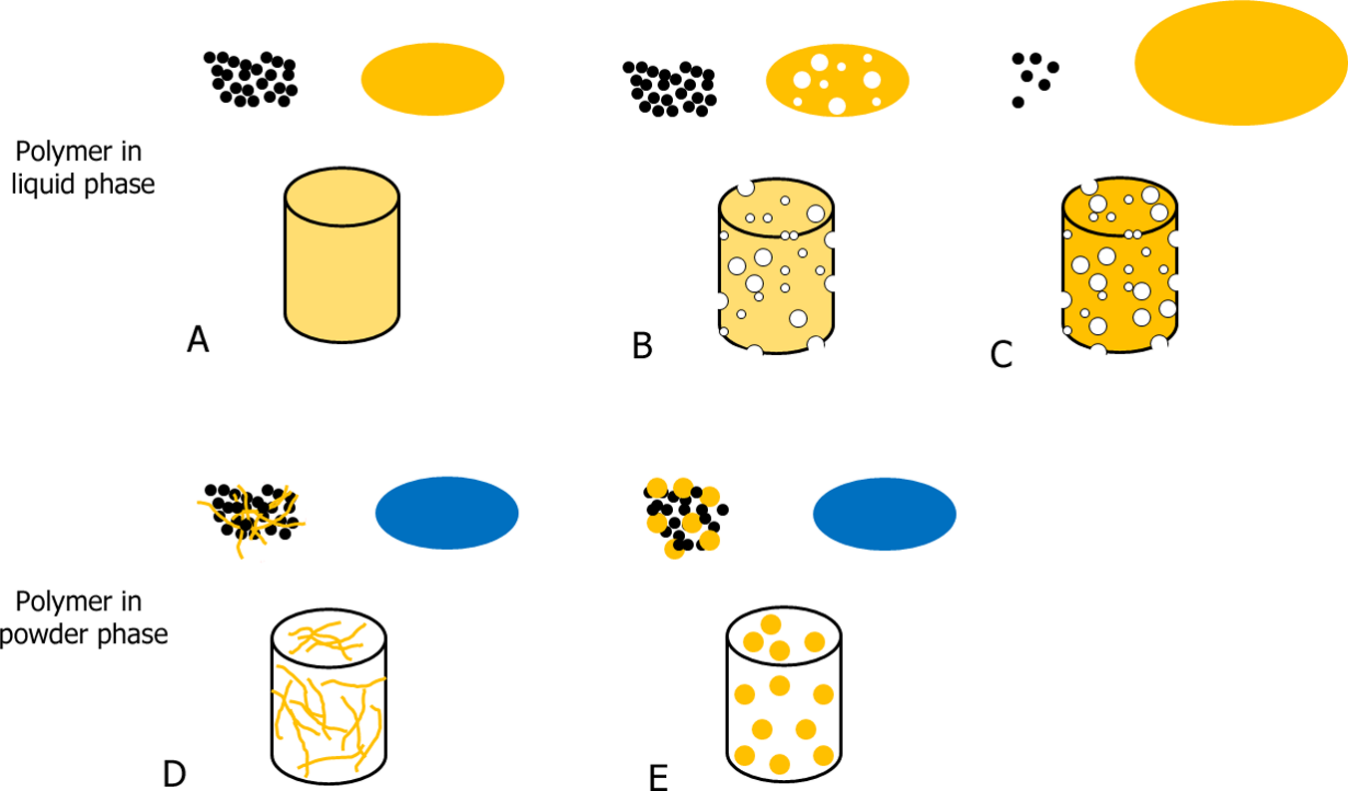
Different strategies for incorporating polymers in CPCs. The polymer can be incorporated either in the liquid phases (A, B, and C) or in the powder phases (D and E). A represents the mixing of a polymeric solution with the CPC powder to obtain a set CPC, which has the polymer homogeneously distributed in the structure. B represents foaming of the liquid solution, which is then combined with the powder to obtain a set macroporous CPC. C represents the incorporation of a small amount of CPC powder in a big volume of polymer solution, upon which a slurry is formed and is then freeze–dried, resulting in a macroporous polymer–CPC scaffold. D represents the combination of the powder phase with polymer fibers to obtain a fiber-reinforced CPC. Moreover, the fibers may act as pore generators when degraded. E represents the combination of the powder phase with polymer MSs, which can act as controlled drug eluting systems and simultaneously generate macropores in the CPC. CPC: calcium phosphate cement.

CPCs are composed of a powder phase and a liquid phase. Therefore, polymers can be added to CPC, either dissolved in the liquid phase or in a solid state as an additive to the powder phase, as shown schematically in Figure 1. Obviously, only water-soluble polymers can be added to the CPC liquid phase. In this case, the polymer will be present as a continuous phase throughout the entire CPC and, what is more important, the solubilized polymer will be able to interact with the cement setting reaction, namely the dissolution of the original phase and the precipitation of the final product. Depending on the final CPC properties desired, the liquid phase properties may be altered by changing several features of the polymer, such as concentration, molecular weight, and polymer chain length. Conversely, when the polymers are added in solid form, they will act as a second and discontinuous phase in the cement inorganic matrix. Although the extent of chemical interaction with the setting reaction is expected to be lower, the morphology, size, and percentage of this second phase will have significant effects on the handling properties and on the final performance of the material.

## Polymers incorporated into CPCs and their effects on CPCs properties

This section describes the most significant advances in the development of polymer-modified CPC. The results are classified according to the way of adding the polymer within the CPC, and in function of the origin of the polymer, namely, natural or synthetic. Further details of the different formulations are summarized in Table 2.

**Table 2.** Description of the different natural and synthetic polymers incorporated into the liquid or the powder phase of the CPC

Polymer name	% Weight/specifications	Liquid phase	CPC composition	CPC end product	L/P ratio	Main effect	References
Liquid phase							
Natural polymers							
Gelatin	0%–20%	H_2_O or Na_2_HPO_4_ solution	α-TCP	HA	0.40–0.80	Foaming of the gelatin solution results in injectable self-setting gelatin–HA foams	23, 25
	5%	10× PBS	α-TCP	HA	1.2	Increase in initial cell adhesion and proliferation	37
	0%–10%	H_2_O	CaCO_3_–MCPM	HA	0.55	Increase in setting time. Small effect on cell proliferation. Decrease in mechanical properties	38
	15%	H_2_O	α-TCP–DCPD	HA	0.3	Similar proliferation values, but enhanced primary osteoblast activation and ECM mineralization process	39
	2%–10%	H_2_O	CaCO_3_–MCPM	HA	0.4	Increase in setting time. Higher mechanical properties for lowest gelatin concentration (2%)	40
	20%	H_2_O	ACP–DCPD	HA	—	Increase in setting time. Decrease in mechanical properties	41
	0%–20%	H_2_O	α-TCP–DCPD	HA	0.3	Faster final production. Increase in mechanical properties with increase in gelatin concentration	42
	10%	H_2_O	α-TCP–DCPD	HA	0.3–0.4	Increase in compressive strength	43
	0%–20%	H_2_O	α-TCP	HA	0.28–0.5	Increased mechanical properties when gelatin concentration up to 5%	44
Collagen	0%–5%; fibers (∅ = 0.1–3 µm; L = 20–100 µm)	H_2_O	TTCP–DCPA	HA	0.25–0.4	Increase in cell adhesion. Decrease in compressive strength as collagen percentage was increased	45
	3%	100–800 mM citric acid	MCPM–β-TCP	DCPD	0.29	Increase in cell adhesion. Mechanical properties maintained similar to control	46
	0%–2%	H_2_O/0.2 M N_2_HPO_4_	TTCP–DCPA	HA	0.29	Increase in setting times. Decrease in compressive strength as collagen percentage was increased	47
Chitosan	20%	H_2_O	ACP–DCPD	HA	0.2	Increase in setting times. Decrease in compressive strength	41
	0%–20%	H_2_O	TTCP–DCPA	HA	0.5	Increase in setting time. Increase in flexural strength	48
	40%	Glycerol and Ca(OH)_2_	TTCP–DCPA	HA	0.5	Increase in setting time. Increased antiwashout properties. Increase in diametral tensile strength. No cell cytotoxicity	49
	0%–6%	1 M phosphate buffer	MCPM–CaO	HA	0.44–1.04	Increase in compressive strength for low chitosan percentage. Decrease in compressive strength higher than 3%	50
	0%–15%	1 M Na_2_HPO_4_	DCPD–Ca(OH)_2_	HA	0.44–1.04	Increase in setting times. Increase in compressive strength as chitosan percentage is increased	50
	0%–8%	0.15 g MgCO_3_ + 0.18 mL 30 wt.% H_3_PO_4_	α-TCP or TTCP	HA	0.125	Conversion to HA inhibited by large amounts of chitosan	51
	0%–30%	H_2_O	TTCP–DCPA	HA	0.5	Reduction in setting times. Increase in flexural strength up to 20 wt.% chitosan	52
	0%–15%	H_2_O	TTCP–DCPA	HA	0.22–0.5	Increase in flexural strength. No significant effect on cell activity	53–58
	0%–15%	H_2_O	TTCP–DCPA	HA	0.5	Increase in flexural strength. Significant increase in ALP cell activity	59, 60
	0%–15%	H_2_O	TTCP–DCPA	HA	0.5	Increase in flexural strength	61
	2%	1.5% Acetic acid solution	α-TCP	HA	0.33	Increase in compressive strength. No cell cytotoxicity. Bigger osteoclastic cell morphology	62
	0%–15%	5% Malic and malonic acid	β-TCP, CaO, MgO, ZnO. TTCP–DCPA	HA	0.7	Increase antiwashout properties. No significant effect on injectability	63–67
	0%–12%	1 M Na_2_HPO_4_	MCPM–CaO or DCPD–Ca(OH)_2_	HA	0.96 or 2.29	Negative effect of chitosan on biodegradation	68
	0%–15%	PBS with 0–100 ng/mL protein A solutions	TTCP–DCPA	HA	0.25–0.5	Sustained release of gentamicin	69
Alginate	2.2%	0.2 M neutral phosphate solution	TTCP–DCPA	HA	0.29	Increase in setting time. No significant effect of alginate on compressive strength up to 10% concentration	70
	2%	0.2 M neutral phosphate solution	TTCP–DCPA	HA	0.25	No effect on setting time. Decrease in tensile diametral strength	12
	0%–0.5%	1 M Na_2_HPO_4_	MCPM–CaCO_3_ incorporation of gentamicin 2.5% or 5% in powder	HA	0.45	Slight increase in setting time. Maximum strength unaffected. Extended release of gentamicin	71
	20%	H_2_O	ACP–DCPD	HA	0.2	Increase in setting time. Decrease in mechanical properties	41
	0%–6%	2.5% Na_2_HPO_4_	α-TCP	HA	0.6–0.87	Increase in setting time. Decrease in diametral tensile strength	72
	0%–1%	1% Na_2_HPO_4_	α-TCP, DCPD, CaCO_3_, and PHA	HA	0.35–0.40	Reduction injectability	14
	0%–1%	Chondroitin sulfate and succinic acid	α-TCP–TTCP–DCPD	HA	0.3	Increase in cohesion and antiwashout properties	73
	0%–2%	105 mM CaCl_2_	ACP–DCPD (α-BSM)	HA	0.8	Support cell growth and osteogenesis	74
Hyaluronate	0%–0.5%	0.5 M citric acid	β-TCP–MCPM	DCPD	0.4	Setting times were increased. Mechanical properties unaffected	75
	0%–8%	2.5% Na_2_HPO_4_	α-TCP	HA	0.35	No effect on mechanical properties	76
	0%–1%	0.2 M PBS	TTCP–DCPD	HA	0.35	Increased injectability	77
Cellulose	0%–2.2%	Na_2_HPO_4_	TTCP–DCPA, α-TCP–CaCO_3_, DCPA–Ca(OH)_2_	HA	0.25 and 0.27	Increase in setting time. Increase in mechanical properties	78,79
	0%–3%	0.2 M sodium phosphate	TTCP–DCPA, TTCP–DCPD	HA	0.5	Similar setting times to control. Mechanical properties increased. Increase in injectability	80
Silk	0%–2%	0.9 NaCl solution	ACP–DCPD (α-BSM)	HA	0.8	Decrease in compressive strengths	74
	0%–2%	0.25 M NaHPO_4_/Na_2_HPO_4_	α-TCP	HA	0.4	Increase in flexural strength. No difference in setting time or cell viability respect to control	81
Chondroitin sulfate	0%–20%	H_2_O and 0.5 M citric acid	ACP–DCPD^a^ and β-TCP–MCPM^b^	HA^a^ and DCPD^b^	0.39–0.5	Slightly higher setting times and mechanical properties	41, 82
Chitin	0%–4%	H_2_O	α-TCP–TTCP–DCPD	HA	0.43	Reduction of setting times. Increase in compressive strength	83
Albumen	0%–12%	H_2_O or Na_2_HPO_4_ solution	α-TCP	HA	0.35	Macroporous self-setting calcium phosphate foams are obtained. Faster resorption in vivo	21, 22
Soybean- derived hydrogel	0%–20%	Na_2_HPO_4_ solution with or without gelatin	α-TCP	HA	0.65	Injectable calcium phosphate foams with an enhanced osteoblast adhesion growth	24
Synthetic polymers							
Polyesters and polyethers	0%–20%	2% Alginate in H_2_O	PCCP–DCPA	HA	0.31–1	Scaffold immersed in PLGA solution. Increase in the mechanical properties in the presence of PLGA	84
	0%–3%	PEG in H_2_O	TTCP–DCPA	HA	0.33	Concentrations higher than 1% decreased mechanical properties	85
	1.4% PPF	*N*-vinyl pyrrolidone	TTCP–DCPA	HA	0–1	Decrease in mechanical properties. Prolonged release of protein Rg1	86
	0%–1% Liquid (polysorbate 20)	Glycerol	MCPM–β-TCP	DCPD	0.21–0.44	Since the paste is formed with glycerol, no water is contained and reaction does no start until immersed in water. Setting times and compressive strength similar to control	87
	—	PEG and glycerin	MCPM–β-TCP	DCPD	0.27–0.4	Increased setting times. Higher cohesion and antiwashout properties. Higher inflammatory response than control	88
	0%–0.5% PEG and glycerin	Na_2_HPO_4_ and citric acid	ACP–DCPD	HA	0.5	Decrease in setting times. Reduced injectability	89
	0%–10% Glycerol	Ca(OH)_2_, H_3_PO_4_, and H_2_O	α–TCP and TTCP	HA	0.43	Increase in setting time. Improvement of injectability and reduction in injecting force	90
Polyacrylic acid	0%–1.45%	0.0625 g/mL Gentamicin sulfate	MCPM–β-TCP	DCPD	0.8	Controls the gentamicin release during prolonged time	91, 92
	0%–20% Acrylamide	0.5% MBAM, 0.25% 0.30 mL/g TEMED, 2.5% Na_2_HPO_4_, and 1% PA	α-TCP	HA	0.30–0.32	Significant increase in the compressive and tensile strength. Reduction of the porosity	93
	35% Polymethyl-vinyl ether-maleic acid or 10% polyacrylic acid	H_2_O	TTCP–DCPD–TCP	HA	0.25	Considerable increase in compressive strength, even at short times. Lower cell viability than control after 24 h. After 1 week, similar cell viability to control	94
Fibrin glue	—	Fibrin glue (Hualan Biological Engineering, China)	TTCP–DCPA	HA	0.2–1	Increase in setting times. Considerable increase in compressive strength. No effect on cell proliferation and differentiation after 14-day culture	95
Solid phase							
Natural polymers							
Gelatin MS	0%–10%; MS size 15.48–8.64 µm; bFGF, TGF-β1 and BMP2 incorporated	1% Na_2_HPO_4_	α–TCP–DCPA–CaCO_3_	HA	0.91	Setting time and macroporosity were increased. Compression strength was decreased. Prolonged release of growth factors was obtained	96–98
Gelatin MS	10%; 50–150 µm. Gentamicin incorporated in MS (900 mg)	1% Na_2_HPO_4_	α–TCP–MCPM–CaCO_3_	HA	0.4	Incorporation of MS increased setting times and porosity. Compressive strength was decreased, but could be enhanced by the incorporation of calcium sulfate hydrate. Release of gentamicin can be controlled depending on cross-link of MS	99
Gelatin MS	5%; 20 µg of BMP2 incorporated in implant	1 M Na_2_HPO_4_	TTCP–DCPA	HA	0.45	Release of BMP2 is more prolonged when BMP2 is incorporated in gelatin MS. Can accelerate healing osteoporosis in vivo	100
Gelatin MS	0%–5%	1 M Na_2_HPO_4_	TTCP–DCPA	HA	0.4	The mechanical properties of composite initially increased but decrease with degradation. Increased macroporosity. Optimum amount is 2.5% of mass fraction MS. Good biocompatibility in vitro and in vivo	101
Gelatin MS	48%–57%; size 37 ± 31 µm	2% Na_2_HPO_4_	α–TCP–DCPA	HA	0.35	Adequate degradation in vivo. Increased macroporosity	102
Collagen	0%–5%	H_2_O/0.2 M neutral phosphate	TTCP–DCPA	HA	0.29	Prolonged setting times and reduced mechanical properties	47
Chitosan	0%–2%	H_2_O	ACP–DCPD	HA	0.5	Setting times reduced. No effect on compressive strength	103
Cellulose	0%–6.4%	2.5% NaHPO_4_	α-TCP	HA	0.6–0.87	Increase in injectability	104
Alginate microbeads	1.2% sodium alginate; 0%–70% microbeads; size 207 µm	15% Chitosan in H_2_O	TTCP–DCPA	HA	0.25	Decrease in flexural strength. Cells were able to survive, proliferate, and differentiate	105–108
Alginate	0%–1%	H_2_O	ACP–DCPD	HA	0.5	Setting times decreased. Compressive strength decreased as polymer concentration increased. Injectability reduced	103
Synthetic polymers							
PLGA microspheres	20%; Microspheres 10–110 µm diameter	2% Na_2_HPO_4_ BMP2 adsorbed and entrapped on microparticles	α-TCP, DCPA, and CaCO_3_	HA	0.35–0.5	Controlled degradation of PLGA allows for a prolonged release of the BMP2. In vitro and in vivo were shown to be biocompatible and the presence of the microparticles allowed to obtain interconnected porosity for tissue ingrowth	26, 109–114
PLGA MS	5%; MS size 7–14 µm; gentamicin or BMP2 loading	H_2_O or 4% Na_2_HPO_4_	TTCP–DCPA	HA	0.3	Controlled and prolonged release of growth factor. No change of the setting times or the mechanical performance	115, 116
PGA fibers	0%–45%; fraction volume fiber length 8 mm	15% Chitosan solution or H_2_O	TTCP–DCPA or TTCP–DCPD	HA	0.22–0.4	Material exceeded strength of cancellous bone. Increased flexural strength. Cells presented excellent viability, differentiated, and synthesized bone minerals	117–120
PCL and PLLA fibers	0%–7%; fibers 3 mm	1% Na_2_HPO_4_	α-TCP, DCPA, and CaCO_3_	HA	0.33	Connective channel-like porous structure was created in the CPC. Toughness was improved. Decreased flexural strength	121
PGA fibers	0%–24%; diameter 0.30–0.349 mm	3.5 M H_3_PO_4_ + 100 mM sodium citrate	β-TCP (Plasma Biotal, UK)	DCPD	0.67	The yield and ultimate strength increased. Modulus of elasticity also increased in flexural testing. Regular fiber orientation led to higher mechanical properties compared to random fibers	122
Aramide fibers	0%–9.5%; fraction volume fiber length 3–200 mm	H_2_O	TTCP–DCPA	HA	0.33	Ultimate strength significantly increased. The longer the fibers, the higher the mechanical properties	123
Polyamide fibers	0%–1.6%; diameter 0.1 mm and length 3 mm	2.5% NaHPO_4_	α-TCP	HA	0.55	Increase in compression strength, but was not concentration dependent	124
Polyacrylic acid	0%–25%; 45- to 75-µm particles	H_2_O	TTCP–DCPA	HA	0.4	Increase in setting time proportional to increase in concentration. Significant increase in the compressive strength	125

CPC: calcium phosphate cement; L/P: liquid to powder; TCP: tricalcium phosphate; HA: hydroxyapatite; PBS: phosphate buffered saline; MCPM: monocalcium phosphate monohydrate; DCPD: dicalcium phosphate dehydrate; ECM: extracellular matrix; ACP: amorphous calcium phosphate; TTCP: tetracalcium phosphate; DCPA: dicalciumphoshphate anhydrous; ALP: alkaline phosphatase; PHA: precipitated hydroxylapatite; PCCP: partially crystallized calcium phosphate; PLGA: poly(lactic-*co*-glycolic acid); PEG: polyethylene glycol; PPF: poly(propylene fumarate); MBAM: *N,N*′-methylenebisacrylamide; TEMED: *N,N,N*′*N*′-tetramethylethylenediamide; PA: polyacrylate; MS: microsphere; TGF: transforming growth factor; BMP2: bone morphogenetic protein 2; PGA: polyglycolide acid; PCL: poly(ϵ-caprolactone); PLLA: poly(l-lactic acid); bFGF: basic fibroblast growth factor.

### Polymer addition in the liquid phase

#### Natural polymers

##### Gelatin

Gelatin is a natural polymer derived from collagen, being in fact denatured collagen. Gelatin is soluble in water and shows increased solubility as temperature increases. Gelatin gels at temperatures <38°C–40°C.^126^ The triple helical structure of collagen is degraded, and uncoiled structures are formed during gelatin processing. This results in exposing the RGD sequence found in the triple helical structure of collagen, which is a specific binding amino acid sequence for cells to attach. Therefore, one of the main reasons to incorporate gelatin into a CPC is to enhance cell adhesion. Some studies have shown a positive effect of incorporating gelatin on initial cell adhesion and proliferation,^37,127^ although other studies have reported only a small effect on cell proliferation.^38,39^ Moreover, an increase in the production of bone-related proteins after 3 and 7 days of culture, indicating an increase in osteoblastic activity and differentiation, is observed in gelatin-containing CPCs.^39^ The same authors showed that gelatin stimulated alkaline phosphatase (ALP) activity as well as collagen and transforming growth factor 31 production.^127^ Data indicate that gelatin in CPCs favors osteoblast proliferation and activates their metabolism and differentiation.^127^

Nevertheless, gelatin may negatively affect other parameters, such as setting time, which increases due to the increase in paste viscosity, and subsequent ion diffusion difficulties. Gelatin increases the setting time for a CPC composed of monocalcium phosphate monohydrate (MCPM)-CaCO_3_; this increase is more pronounced as gelatin concentration is increased.^38,40^ The same effect is found for a CPC composed of amorphous calcium phosphate (ACP)–DCPD.^41^ In contrast, the time to completely transform α-tricalcium phosphate (TCP) into calcium-deficient HA was advanced from 7 days in the control CPC to 2 days for the gelatin containing TCP.^42^

Gelatin also affects CPC mechanical properties, although in different ways depending on the amount of gelatin incorporated. Gelatin increases the compressive strength of an α-TCP cement fourfold, which is related to a decrease in sample porosity.^43^ Compressive strength increases linearly as a function of gelatin concentration.^42^ Nevertheless, the general trend is that the highest strengths are obtained with low gelatin concentrations rather than with high gelatin concentrations. Actually, optimum mechanical properties were obtained with a 2 wt.% gelatin solution incorporated into a CPC composed of MCPM–CaCO_3_.^40^ CPCs (both ACP–DCPD and MCPM–CaCO_3_ cements) with gelatin concentrations of 10–20 wt.% clearly showed diminished compressive strength.^38,41^ In contrast, 5 wt.% gelatin was optimum for a α-TCP CPC.^44^ Another study showed no difference due to the presence of gelatin in the CPC.^128^

Gelatin was also used as foaming agent in CPC. Self-setting gelatin–α-TCP foams are obtained by mixing α-TCP with a foamed gelatin solution, which after setting results in a HA solid foam, with high macroporosity and adequate cohesion and injectability.^23,25^

##### Collagen

Collagen is a triple helical structure protein and is the most abundant protein found in bone. Collagen is insoluble in water and requires acidic conditions to solubilize. The presence of collagen has a similar effect to that of gelatin in some cases, which is probably expected, as gelatin is denaturized collagen. Nevertheless, as collagen has a triple helical structure, the RGD sequence is not exposed. Instead, other amino acid sequences are exposed such as the glycine–phenylalanine–glutamine–glycine–glutamic acid–arginine sequence, which may also enhance cell adhesion. The effect of adding collagen on the in vitro biological properties of tetracalcium phosphate (TTCP)–dicalciumphoshphate anhydrous (DCPA), MCPM–β-TCP and α-TCP CPCs was assessed in a cell culture study.^45,46,129^ Initial adhesion was enhanced when the CPC was combined with collagen^45,46^ and so was the proliferation.^129^

Collagen also influenced other CPC properties. When the collagen is incorporated during the liquid phase, the setting times are in the range needed for orthopedic applications, although setting times increase as collagen concentration increases.^47^

Adding collagen decreases the mechanical properties of a TTCP–DCPA CPC, and this decrease is more significant with increasing collagen concentrations.^45,47^ In contrast, the mechanical properties are slightly improved when collagen is incorporated into a brushite CPC.^46^

##### Chitosan

Chitosan is a linear polysaccharide composed of randomly distributed D-glucosamine and *N*-acetyl-D-glucosamine units. Chitosan can clot blood and has antibacterial properties. Chitosan is insoluble in water and soluble under acidic conditions. When incorporated into different CPCs composed of ACP–DCPD, α–TCP, TTCP, MCPM–CaCO_3_, or DCPD–Ca(OH)_2_, chitosan increases setting time and inhibits the setting reaction.^41,48^^–^^51^ Nevertheless, chitosan significantly reduces setting times for TTCP–DCPA cements.^52,130^

The effect of chitosan on flexural and compressive strength has been widely studied. Chitosan increases the flexural strength of a chitosan–CPC composite composed of TTCP–DCPA considerably, and the highest value was reached when 15–20 wt.% chitosan was incorporated into the CPC,^48,53,54,59,61,117^ although optimum results also occurred when CPC–chitosan is synergistically combined with Vicryl fibers or alginate microbeads.^105,117^ Another approach, which actually does not incorporate the polymer in the liquid phase or in the powder phase but in the CPC paste, being the only report that has shown such methodology, also reported an increase in the flexural strength of the composite.^131^ Even though this last work does not correspond to any of the two sections (polymers incorporated into the liquid phase or in the powder phase), it was incorporated into this section since it is the only case reported and because it shows similar trend to the works in which chitosan was incorporated into the liquid phase. In general, flexural strength decreases when the amount of chitosan increases >20 wt.%.^52,55,56^ Similarly, compressive strength drastically decreases when chitosan increases to >10 wt.%.^41,50^ Nevertheless, the compressive strength of CPC composites containing chitosan generally increases.^50,62^ An interesting property of chitosan is its ability to increase the antiwashout resistance of CPC^63–67^ but not injectability.^63–67^

Adding chitosan has a moderate effect on the cell response to CPC. No cytotoxicity^38,49,55–58,62,131^ is found in chitosan-containing CPCs. Moreover, ALP activity increases considerably in the presence of chitosan when mesenchymal stem cells are cultured on a TTCP–DCPA CPC composite containing 15 wt.% chitosan.^59,60^ The same authors reported a similar ALP value for the same chitosan–CPC composite compared to the control CPC when culturing MC3T3-E1 cells.^53^ Cells also survive when encapsulated in sodium alginate droplets and combined with a CPC paste containing chitosan.^57,132^ When preosteoclastic cells are cultured on a CPC containing chitosan, cell morphology and tartrate-resistant acid phosphatase (TRAP) activity are similar to a control CPC, although the osteoclasts are larger.^56,133^

Two different chitosan-containing CPCs composed of either MCPM–CaO or DCPD–Ca(OH)_2_ have shown a lower biodegradation in the presence of chitosan.^68^ The effect of chitosan on the protein release properties of a CPC loaded with protein A has also been studied. Incorporating chitosan results in sustained release when both the amount of chitosan incorporated and the L/P ratio of the composite are adjusted.^69^

##### Alginate

Alginate is an anionic polysaccharide found in brown algae cell walls. It is capable of absorbing 200–300 times its own weight in water and creating a viscous gum. It is known as a biocompatible material, and one of its main features is that it gels through chelation with divalent cations. Alginate has been used for cell immobilization or encapsulation.

When DCPA–TTCP is used as the CPC powder phase, incorporating sodium alginate clearly increases the setting times, and this increase is dose dependent.^70^ Similar results are found for CPCs composed of MCPM–CaCO_3_ combined with alginate.^71^ Nevertheless, this increase in the setting times was only observed when the amount of sodium alginate was >2 wt.%.^12^ A CPC composed of ACP–DCPD also showed increased setting time in the presence of sodium alginate.^41^

The compressive strength of a CPC composed of DCPD–ACP containing sodium alginate decreases as the concentration of polymer increases.^103^ This was also observed for TTCP–DCPA and α-TCP cements, in which the incorporation of low amounts of sodium alginate decreases diametral tensile strength.^12,72^ Nevertheless, diametral tensile strength is not affected by incorporating sodium alginate at up to 10 wt.% into TTCP–DCPA cement.^70^ Accordingly, another study reported a decrease in mechanical properties when the amount of alginate incorporated is 20 wt.%.^41^

Similar to chitosan, sodium alginate hinders the CPC setting reaction and, therefore, delays HA formation. A reduction of injectability has also been reported for sodium alginate-containing cements.^14,103^ Nevertheless, the presence of sodium alginate generally increases the antiwashout properties of the CPC and their cohesion.^73^

Sodium alginate has little effect on cell proliferation and differentiation of human bone marrow-derived mesenchymal stem cells.^74^

##### Hyaluronate

Hyaluronate is an anionic nonsulfated glycosaminoglycan that is biocompatible and may be cross-linked to produce hydrogels. The molecular weight of the polymer is very important when combining hyaluronate with CPC. The setting times of a CPC composed of β-TCP–MCPM increase with increasing hyaluronate concentration dissolved in the liquid phase, as long as the molecular weight is low (300 and 750 kDa), whereas the values are unaffected in the presence of higher molecular weight hyaluronate (1640 kDa).^75^

Hyaluronate incorporated into a α-TCP CPC does not significantly affect the mechanical properties of the composite.^75,76^ In contrast, sodium hyaluronate has high viscosity and creates a network with Ca^2+^ when incorporated into the CPC liquid phase, which increases injectability of the paste.^77^

Adding hyaluronic acid slightly delays new bone formation in vivo, although the response is dependent on the initial composition of the CPC solid phase.^134^

##### Cellulose

Cellulose is a polysaccharide of several hundreds of β(1→4) linked d-glucose units. Cellulose is found in the cell walls of green plants and algae. Variations in the monomers may change the structure of cellulose, hydroxylation forms hydropropyl methylcellulose (HPMC), and the substitutions with carboxyl groups form carboxy methylcellulose (CMC).

Adding HPMC (0–4 wt.%) to a CPC generally increases setting time^78,79^ of α-TCP–CaCO_3_, DCPA–Ca(OH)_2_, and TTCP–DCPA. Nevertheless, values similar to control CPC values have been reported^80^ in the compositions of TTCP–DCPA and TTCP–DCPD cements when HPMC was incorporated (0–3 wt.%).^80^

The mechanical properties (e.g. diametral tensile strength, compressive strength, and elastic modulus) for different CPCs composed of α-TCP–CaCO_3_, DCPA–Ca(OH)_2_, TTCP–DCPA, and TTCP–DCPD tend to increase as the amount of HPMC increases.^78–80^ Nevertheless, opposite results have also been reported, in which the modulus was reduced with added HPMC in a CPC composed of ACP–DCPD.^73^ HPMC drastically increases the injectability of the CPC even at low concentrations, and the injectability tends to increase as polymer concentration is increased for TTCP–DCPA, TTCP–DCPD, and α-TCP compositions.^80^

Adding CMC to the CPC does not significantly improve the in vitro biological properties such as cell proliferation or differentiation.^74^ HPMC has also been used for drug delivery applications. The amount of gentamicin released from a CPC composite made of β-TCP–MCPM and HPMC is reduced, probably due to chemical binding between the polymer and the antibiotic.^91^

##### Others

Other natural polymers have also been combined with CPCs, but only a few studies have been reported. For example, silk reduces maximum compressive strength and the elastic modulus compared to those in a control CPC.^74^ Nevertheless, flexural strength increases significantly in the presence of silk (0.5, 1, and 2 wt.%).^81^ Setting times do not vary in the presence of silk fibroin.^81^ Silk can also be used to increase CPC cohesion.^135^ No differences in terms of cell viability compared to the control CPC were observed when silk was incorporated.^81^ Another example is incorporating starch and chondroitin sulfate into a CPC, which results in slightly higher setting times and mechanical properties compared to those of a control CPC.^41,82,103^ Albumen and soybean have also been incorporated into CPCs, with the purpose of creating a liquid phase foam, which enables the production of a macroporous injectable CPC.^22,24^

Finally, chitin has been incorporated into a CPC composed of α-TCP–TTCP–DCPD at 4 wt.% chitin, resulting in reduced setting times from 32 min in the control to 14 min for the composite CPC.^83^ Incorporating chitin also increases compressive strength from 23 MPa in the control to 33 MPa in the composite material.^83^ However, a high chitin content is detrimental to CPC resorption under in vivo conditions.^136^

#### Synthetic polymers

##### Polyesters and polyethers

Polyesters are thermoplastic polymers that contain an ester functional group in their main chain. They are degradable, and the degradation rate is highly dependent on composition. Although hydrolytically degradable, they have far lower water absorption and shrinkage than those of natural polymers. While poly(ϵ-caprolactone) is highly flexible, polylactide acid (PLA) and polyglycolide acid (PGA) have relatively high strength and elastic modulus. Therefore, one of the possible main functions of the polyesters in CPCs is to increase mechanical strength. However, these polymers are not water soluble, and therefore, they cannot be directly incorporated into the liquid phase of the CPC. With this in mind, poly(lactic-*co*-glycolic acid) (PLGA) dissolved in dichloromethane was infiltrated into the macropores of a alginate/CPC scaffold. Incorporating PLGA in CPC at a concentration of 20 wt.% increased significantly the mechanical strength.^84^ Opposite results were observed when water-soluble copolymers were obtained by combining polyethylene glycol (PEG) with poly(γ-benzyl l-glutamate), poly(γ-ethyl l-glutamate), and poly(γ-methyl l-glutamate) and incorporated into a CPC. As a result, mechanical strength decreased when polymer concentration was >1 wt.%.^85^ The incorporation of poly(propylene fumarate) (PPF) also decreases the mechanical strength of a CPC as the amount of PPF is increased in the composite.^86^ The combination of a CPC composed of TTCP–DCPA with PPF resulted in prolonged release of protein Rg1 with complete release over 20 days without regard to the protein content incorporated.^86^

PEG is a polyether composed of glycerol monomers. It has been used to obtain premixed CPCs. In fact, when the monomers are combined with CPC, water-free pastes are formed, which can be stored for a long period without reacting. This means that CPC pastes can be prepared at the bench and stored until needed (e.g. operating theater).^49,78,87,137^ When the premixed CPC pastes are immersed in water for the reaction, they present setting times and compressive strength similar to the conventional CPC.^87^ However, the mechanical strength decreases in the presence of PEG after a 7-day reaction in water,^88^ and the injectability of the CPC pastes is reduced with added PEG, glycerol, and glycerin.^89,90^ Contradictory results were found for setting times; the presence of glycerin and PEG decreases the setting times of ACP–DCPD cement.^89^ Although PEG increases the setting times of β-TCP–MCPM CPC,^88^ it is known as an effective antiwashout agent.^88^

##### Polyacrylic acid

Polyacrylic acid (PAA) and its derivatives are capable of absorbing water many times their weight. At neutral pH, PAA loses protons and is then negatively charged, facilitating the combination with a range of antibiotics or similar drugs for sustained release. When gentamicin sulfate is incorporated into CPC modified with PAAs, the antibiotic shows quite sustained release from the cement composite.^92^ The final amount of released gentamicin was thus reduced in the PAA–CPC composites.^91^ Nevertheless, one of the main problems is that the reaction is hindered, as few reactions occur in the presence of PAA even after 1 month.^128^

Polyacrylates have considerable effects on mechanical properties. Compressive strength increases substantially to 55 MPa when ammonium PAA is incorporated into the CPC, which is contrasted with 25 MPa for a CPC without PAA.^93^ The increase in compressive strength can also be deduced from the reduction in composite porosity.^93^ Furthermore, adding PAA allowed the brittle CPC to become more ductile.^138^ Within the same family of polymers, a 35 wt.% aqueous solution of poly(methyl vinyl ether-maleic acid) and 10 wt.% PAA were added to a CPC powder composed of 60 wt.% TTCP, 30 wt.% DCPD, and 10 wt.% TCP. As a result, mechanical properties increased considerably with respect to the control CPC, reaching ~70 MPa after 2 weeks compared to ~18 MPa in the control CPC. Moreover, 70 MPa is achieved in as short as 30 min in a CPC containing 10 wt.% PAA.^94^ TTCP–DCPA CPC also shows a significant increase in strength (diametral tensile strength and compressive strength) with added poly(methyl vinyl ether-maleic acid).^139^

Incorporating PAA and poly(methyl vinyl ether-maleic acid) results in lower cell viability than that in a control CPC composed of TTCP–DCPD–TCP after the initial 24 h; however, cell viability recovered to a level higher than that of the control CPC after 1 week.^94^ In general, lower cytotoxicity is achieved when CPCs are combined with PAA and derivatives compared to the acrylic bone cements.^140^ Composite CPCs have proven in vivo tissue compatibility, suggesting possible clinical applications.^141^

##### Fibrin glue

Fibrin glue is produced as a reaction product of fibrinogen with thrombin and is used to create a fibrin clot. Fibrin glue significantly increases setting times of a CPC composed of TTCP–DCPA powder.^95^ The presence of fibrin glue also increases compressive strength significantly.^95^ Nevertheless, incorporating fibrin glue into TTCP–DCPA cement does not have a significant effect on the cell proliferation or ALP activity after 14 days of culture.^95^ Furthermore, no significant difference in bone formation is observed due to the incorporation of fibrin glue.^142^

### Polymer addition as a solid phase

The addition of polymers in solid state, as a second phase in the CPCs, is aimed at achieving two main objectives. On one side, to act as a reinforcing phase that enhances the mechanical properties of the CPC and on the other side to create macroporosity in the CPC after dissolving the polymer, which promotes tissue colonization and eventually enhances CPC resorption. Polymers can be added in the form of powders, MSs, or fibers.

#### Natural polymers

Adding natural polymers in the form of a liquid phase is preferred, as explained in the previous section, because they dissolve well in water-based liquid. However, the solid forms of natural polymers, such as MS, fibers, and powders, have also been studied. Gelatin MSs have been incorporated into CPC powder to stimulate the degradation and resorption of a CPC.^96–102,109^ Different amounts and sizes of gelatin MSs were added to the powder phase of the CPC, which was then mixed with the liquid phase. As a result, the MSs degraded in water with time to provide space for cells to penetrate and for new bone ingrowth. Growth factors can be loaded for therapeutic applications.^98,100^

Lyophilized collagen has also been incorporated as the solid phase. However, this results in significant difficulty in mixing and retards the setting reaction.^47^ Chitosan has also been incorporated into the solid phase of a ACP–DCPD cement, and setting times are reduced, but no effect on compressive strength was observed.^103^ The injectability of an α-TCP cement was increased when cellulose was incorporated into the powder phase of the cement.^104^

Alginate has been added to a CPC (TTCP^–^DCPA) in the form of microbeads,^54^ resulting in an increase in the mechanical properties. Moreover, the presence of alginate microbeads helped the formation of macrochannels in the CPC, which stimulate vascularization in vivo and help biodegradation of material.^143^ In contrast, if alginate is added as smaller particles as the powder phase, setting times decrease, and the compressive strength and injectability also decrease.^103^

Because of the specific property of sodium alginate for encapsulating tissue cells within gelled microcapsules,^54,106^^–^^108^ cell-encapsulating alginate beads have also been added to CPCs. Cells inside the alginate beads are viable and undergo appropriate cellular processes, such as cell mitosis and tissue differentiation. Therefore, a combined system composed of CPC–alginate with cells is considered a possible tissue-engineered construct. However, concerns may remain as to the mechanical properties of the CPC.

#### Synthetic polymers

Compared to natural polymers, the synthetic polymers are added more preferably in the form of a solid phase, which is mainly due to the difficulty in dissolving synthetic polymers in water-based liquids. The well-known degradable copolymer PLGA has been widely used as a second solid phase of CPCs to deliver growth factors and antibiotics in a sustained and controllable manner.^110,111,115,116^ However, even though the growth factor is released from the polymer, this can also be adsorbed on the surface of the CPC due to its high affinity for proteins, this resulting in a reduction of the final release rate.^144^ As these degradable biopolymers added to CPCs have already proven to be biocompatible,^112^ more attention has been given to the control of degradation rate and obtaining highly interconnected macroporosity.^26,109,113,114^

The fiber form of synthetic polymers has been incorporated most widely because of the beneficial mechanical properties of the fibers, such as tensile strength and elastic modulus.^30,105,117,118^ In general, when polyester fibers were incorporated, the flexural strength and work of fracture increased considerably, and the behaviors were greatly dependent on the fiber length and diameter.^27,105,119–122,145^ Of special interest is the incorporation of aramide fibers, which have extremely high flexural strength and work of fracture compared to any other types of polymers, including PGA.^123,146^ In contrast, adding polyamides such as nylon has no significant effect on the mechanical properties of α-TCP cement.^124^ It was also shown that electrospun submicron fibers enhanced mineralization behavior of cells cultured on the CPCs, which was attributed to the higher surface area and some possible biomimetic features of the fiber morphology.^121^ The fiber form of degradable polymers also generates pores during degradation within the cement.^147^ Acrylate derivatives incorporated into CPC also showed increased setting time, and the increase was proportional to the polymer concentration.^125^ The compressive strength was also considerably increased.^125^

## Conclusion

The combination of polymers with CPC, either solubilized in the liquid phase or as a second solid phase, has proven to be an interesting strategy for the development of bone substitutes with improved performance. Whereas CPCs have outstanding biocompatibility and osteoconductivity, they also have some intrinsic limitations that can be counteracted by the incorporation of a polymer in their formulation. The range of properties that can be modified by the addition of a polymer is broad, covering aspects as diverse as the rheological or the mechanical behavior, the rate of resorption, or the cell and tissue response. The large number of publications on this subject demonstrates, from different perspectives, the feasibility of this approach. However, there are still many areas for further work, especially in terms of understanding and controlling the interactions between the organic and inorganic phases, which may open new avenues for the development of novel self-assembled materials through biomimetic routes. Furthermore, the biological behavior of the CPCs can still be further improved and in this sense, incorporation of other molecules, such as growth factors or genes, can overcome some of the limited biological functionalities. Therefore, the incorporation of these different types of polymers may be a useful tool to be able to control the delivery of the different biologically active molecules.
